# Supplementary material for: Non-trivial surface states of samarium hexaboride at the (111) surface
Source: Nat Commun. 2019 May 24;10:2298. doi: 10.1038/s41467-019-10353-3 (PMC6534584; doi:10.1038/s41467-019-10353-3)
Supplement: Supplementary file 1 — Supplementary Information [file 41467_2019_10353_MOESM1_ESM.pdf]

# Supplementary Information for: Non-trivial surface states of samarium hexaboride at the (111) surface

Yoshiyuki Ohtsubo,[1,2,*] Yuki Yamashita,[2] Kenta Hagiwara,[2] Shin-ichiro Ideta,[3] Kiyohisa Tanaka,[3] Ryu Yukawa,[4] Koji Horiba,[4] Hiroshi Kumigashira,[4] Koji Miyamoto,[5] Taichi Okuda,[5] Wataru Hirano,[6] Fumitoshi Iga,[6] and Shin-ichi Kimura[1,2,†]

[1]*Graduate School of Frontier Biosciences, Osaka University, Suita 565-0871, Japan*

[2]*Department of Physics, Graduate School of Science, Osaka University, Toyonaka 560-0043, Japan*

[3]*Institute for Molecular Science, Okazaki 444-8585, Japan*

[4]*Photon Factory, Institute of Materials Structure Science, High Energy Accelerator Research Organization (KEK), 1-1 Oho, Tsukuba 305-0801, Japan*

[5]*HiSOR, Hiroshima University, Higashi-Hiroshima 739-0046, Japan*

[6]*College of Science, Ibaraki University, Mito 310-8512, Japan*

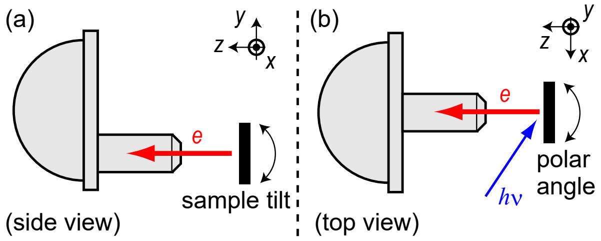


Supplementary Figure 1: Schematic drawings of angle-resolved photoelectron spectroscopy (ARPES) and spin-resolved ARPES geometry.

**Supplementary Note 1: Experimental geometry of angle-resolved photoelectron spectroscopy (ARPES) and spin-resolved ARPES (SARPES)**

Supplementary Figure 1 shows the geometry of ARPES and SARPES setup in this work. All of the geometries used in this work at UVSOR-III, Photon Factory, and HiSOR were nearly the same with only a small quantitative difference: photon-incident angles with respect to the normal of the electron analyser ranged from 45 to 50°. A hemispherical electron analyser with 2D electron detector detects a range of photoelectrons whose emission angles lie in the $xz$ plane in Supplementary Figure 1. The sample was tilted to observe the 2D Fermi contour (FC), as shown in Supplementary Figure 1 (a). The photon-incident plane for Figs. 2 and 3 in the main text is slightly ($\sim$10°) away from the high-symmetry ($\bar{1}10$) plane. Thanks to this geometry together with off-normal-incident circularly polarized photons, no symmetry operation in the photoexcitation matrix element vanishes the photoelectrons from the surface states in Figs. 2 and 3 in the main text.

In the SARPES measurement, only the photoelectrons normal to the electron analyser (red arrows in Supplementary Figures 1 (a) and 1 (b)) are detected with spin separation by very low energy electron diffraction (VLEED) spin detector [1]. The tilt and azimuthal angles (sample rotation around the $z$ axis) are used to change the in-plane wave vector of each SARPES spectra shown in Fig. 4 in the main text. The SARPES setup used in this work has two independent VLEED targets whose surfaces are normal to the $x$ and $y$ axes in Supplementary Figure 1, respectively [2]. For the spectra shown in Fig. 4, the latter target, resolving the spin polarization along $x$ and $z$ in Supplementary Figure 1, was used. The $x$ orientation is parallel or anti-parallel to $[1\bar{1}0]$ or $[\bar{1}\bar{1}2]$ depending on the sample azimuthal angle. The $z$ orientation is nearly parallel to the surface normal of the

sample. Along the polar angle, transition matrix elements are different for the positive and negative emission angles resulting in the different photoelectron intensities as well as spin polarisations [3]. Such artificial asymmetry from the experimental geometry is cancelled out in the spin-resolved spectra by using the tilt angle. For the spin-resolved spectra in Supplementary Figure 6, the polar angle (sample rotation around the $y$ axis) was used with the other VLEED target whose surface is normal to $x$, resolving the photoelectron spins along $y$ (parallel/anti-parallel to $[1\bar{1}0]$ in the geometry for Supplementary Figure 6) and $z$ (along [111]).

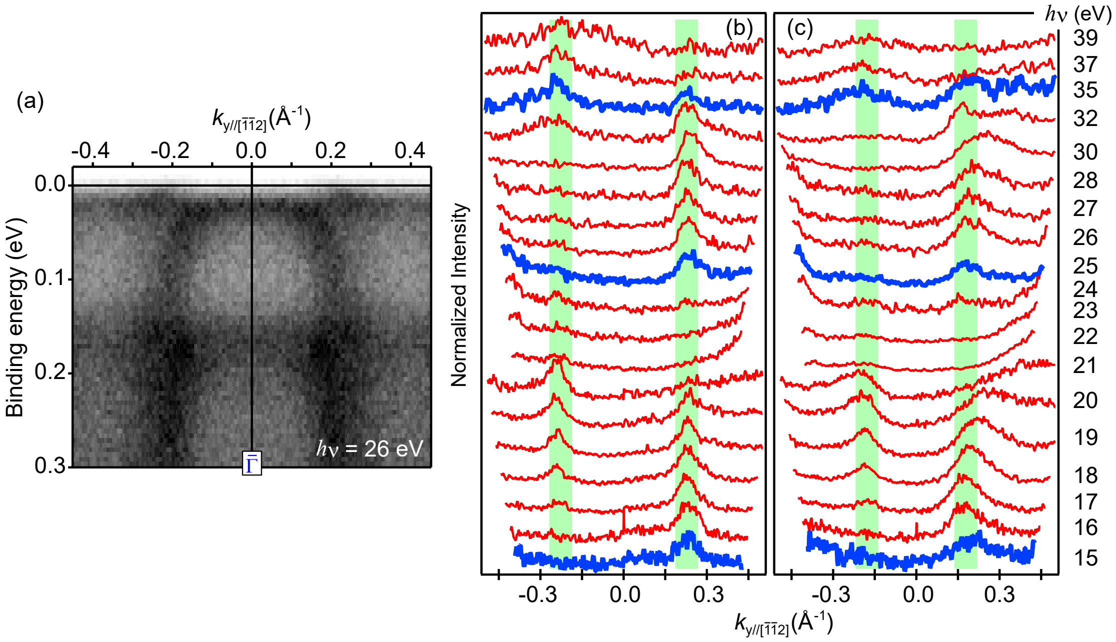


Supplementary Figure 2: (a) ARPES intensity plot taken with linearly polarized photons ($h\nu$ = 26 eV) along $\bar{\Gamma}-\bar{\mathrm{M}}$ at 15 K. The ARPES intensities are symmetrized with respect to $\bar{\Gamma}$. (b, c) APRES momentum distribution curves (MDCs) along $\bar{\Gamma}-\bar{\mathrm{M}}$ at 15 K, taken with linearly polarized photons at photon energies from 15 to 39 eV. The binding energies are at (b) the Fermi level (0 meV) and (c) 60 meV. The energy windows are 10 meV. Green fat lines are the guides to the eye. The triangles in (c) represents the humps away from the green lines.

**Supplementary Note 2: Photon-energy dependence of the bands around the Fermi level**

Supplementary Figure 2 (a) shows the ARPES intensity plot taken with 26 eV photons, different from those shown in Figs. 2 and 3 in the main text. It shows nearly the same dispersions of surface bands, $S1$, $S2$ and $F$, as those taken with 35 eV photons, suggesting their 2D origin. To make the comprehensive analysis, incident-photon-energy dependent momentum distribution curves (MDCs) at the Fermi level and 60 meV (crossing $S2$) are shown in Supplementary Figures 2 (b) and 2 (c), respectively. As guided by the fat lines, it is clearly shown that the peak positions of $S1$ show no change depending on the incident photon energies. The case is similar for $S2$ with the peaks staying in the fat lines. However, one can find broad features away from the fat line, indicating the $k_\mathrm{z}$ dispersion. They would be from the Sm-5$d$ bulk bands. The energy range checked here, 15 to 39 eV, corresponds to 1.1 Å$^{-1}$ along the surface normal, assuming the inner potential of 10 eV (this is a typical value of the inner potential for the calculation of the wave vector along the surface normal). While 1.1 Å$^{-1}$ is ∼80 % of the wavenumbers between Γ and R in the bulk BZ, it could be reduced to 40 % if the inversion at Γ is assumed. From this $k_z$ range, we cannot conclude if $S_2$ is independent from the bulk bands found above or $S_2$ itself is a part of such $k_z$ dispersion. The additional observation with even wider $k_\mathrm{z}$ range is difficult, because the intensities of $S1$ and $S2$ decreases drastically in the photon energy range away from what is shown here. It would be due to the photoexcitation cross section. Based on this analysis, we discussed two alternative scenarios for $S_2$; as a surface resonance or a part of bulk Sm-5$d$ bands in the main text.

**Supplementary Note 3: Band dispersion around the Fermi level along $\bar{\Gamma} - \bar{\mathrm{K}}$**

Supplementary Figure 3 shows the band dispersion of $SmB_6$(111) along $\bar{\Gamma} - \bar{\mathrm{K}}$, traced by the ARPES energy and momentum distribution curves. Similar to those along $\bar{\Gamma} - \bar{\mathrm{M}}$ (Fig. 3 in the main text), there are metallic state crossing the Fermi level at ∼0.25 Å$^{-1}$ and 0.7 Å$^{-1}$, nearly localized state around 0.03 eV, and the other dispersive bands between 0.03 to 0.18 eV, as indicated by the peaks overlaid on the 2D ARPES image (Supplementary Figure 3 (a)).

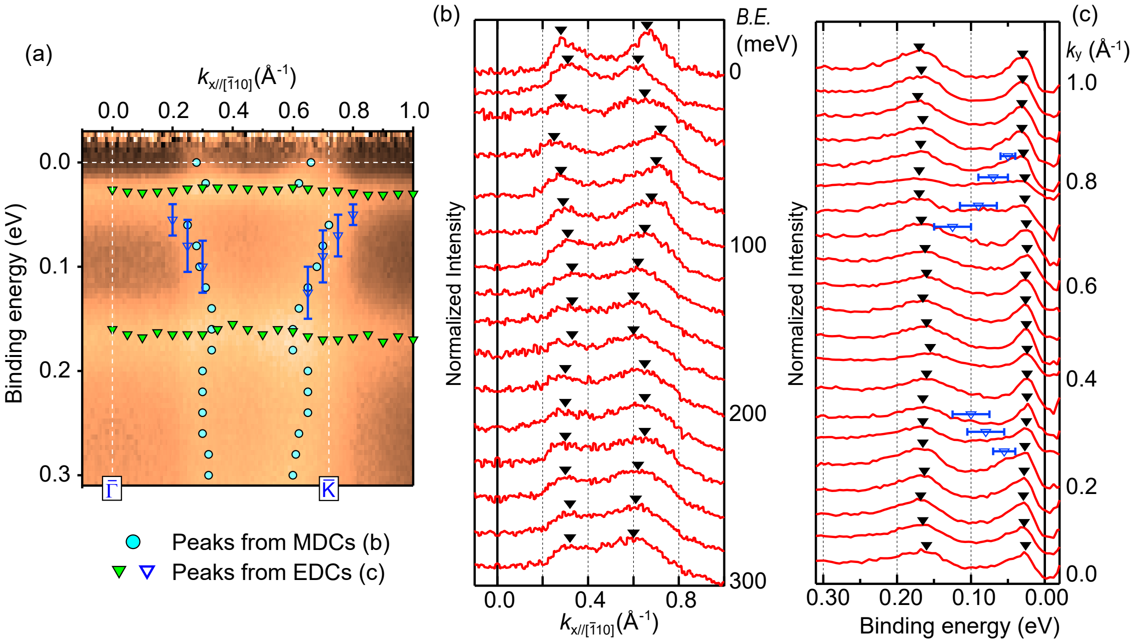


Supplementary Figure 3: (a) ARPES intensity plot taken with circularly polarized photons ($h\nu$ = 35 eV) along $\bar{\Gamma} - \bar{\mathrm{K}}$ at 15 K. (b, c) ARPES (b) MDCs and (c) EDCs taken from Supplementary Figure 3 (a). The filled triangles in (b, c) indicate the peak positions. The open triangles with error bars in (c) are the energy positions of broad features. The width of the bars are explained in the text.

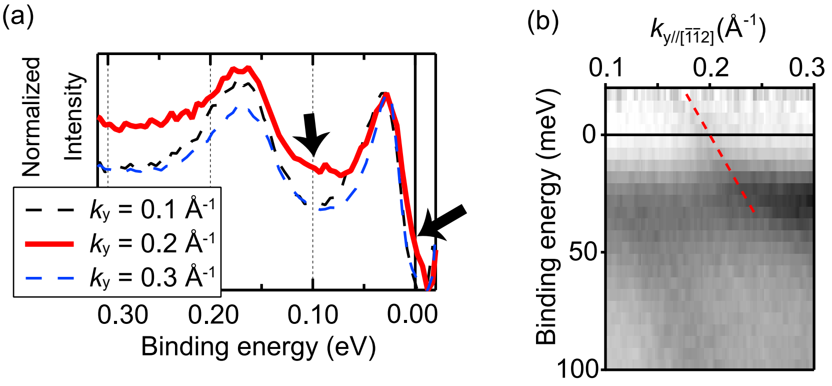


Supplementary Figure 4: (a) ARPES EDCs along $\bar{\Gamma} - \bar{\mathrm{M}}$ (the same data as shown in Fig. 3 (c) in the main text) overlapped with each other. (b) ARPES close-up image along $\bar{\Gamma} - \bar{\mathrm{M}}$ around $k_{\mathrm{F}}$, taken from the same data as Fig. 3. A dashed line is the guide of the Fermi velocity (∼0.8 eV Å).

**Supplementary Note 4: Additional data for ARPES analysis along $\bar{\Gamma}-\bar{\text{M}}$**

On the EDCs, it is difficult to trace the bands with steep dispersion, as $S1$ and $S2$ along $\bar{\Gamma}-\bar{\text{M}}$. However, they actually appear as broad features on EDCs. To show it clearly, Supplementary Figure 4 (a) shows the EDCs at three $k_y$ points along $\bar{\Gamma}-\bar{\text{M}}$; each spectrum is normalized by the peak height at ~0.03 eV. As indicated by the allows, the photoelectron intensities at the Fermi level and ~0.1 eV at $k_{\text{y}} = 0.2$ Å$^{-1}$ are higher than the others; they correspond to $S1$ and $S2$.

The Fermi velocity of $S1$ is an important parameter to be compared with those obtained by the other methods. For a reference, we estimated the Fermi velocity. Around the Fermi level, the dispersion of $S1$ is nearly linear as the dashed guide in Supplementary Figure 4 (b). Based on this slope, the Fermi velocity for $S1$ along $\bar{\Gamma}-\bar{\text{M}}$ is estimated to be 0.8 eV Å.

**Supplementary Note 5: Sizes of FCs obtained by ARPES**

The Fermi surfaces of other $R$B$_6$ materials ($R$: a rare-earth element) with the same CsCl-type lattice as SmB$_6$ are already known, such as those of CeB$_6$ [4, 5], where the Fermi surface is the oval ones touching with each other. The FCs of the topological surface states of SmB$_6$(111) observed here is larger than them, showing overlaps along $\bar{\Gamma}-\bar{\text{K}}$. It means the FCs of valence-fluctuated SmB$_6$, whose valence of Sm is around 2.6, is larger than that of CeB$_6$ with trivalent Ce. In order to examine the role of $c$–$f$ hybridisation to the size of FCs, the size of the constant-energy contour below the hybridization energy (~30 meV) is shown in Supplementary Figure 5 (a). The size of the contour is slightly larger than that of FC ($k_y = 0.17$ Å$^{-1}$, ~5 % larger semi-major axis) and this change is isotropic to any orientations. Since the dispersion of $S2$ at 100±40 meV from MDCs are nearly linear, we could extrapolate them to estimate a size of a virtual FC without $c$–$f$ hybridization, as shown in Supplementary Figure 5 (b). The $k_{\text{F}}$ shifts to 0.11 Å$^{-1}$ (-0.08 Å$^{-1}$ from the actual $k_{\text{F}}$). Although this shift is slightly smaller than that of SmB$_6$(001) (~0.12 Å$^{-1}$) [6], this shift causes even larger overlap of FCs.

The overlap of topological surface states (TSS) could cause interesting electronic phenomena. In topological crystalline insulators (TCIs), the Lifshitz transition accompanied by a Van Hove singularity from the overlapped double topological Dirac cones is expected

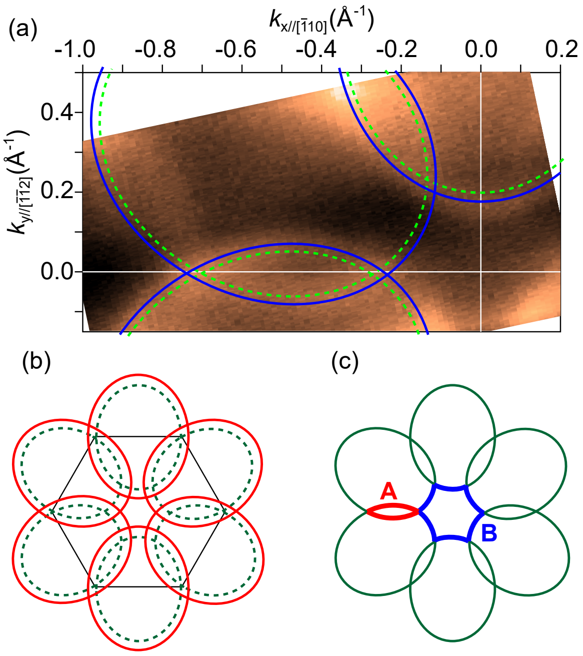


Supplementary Figure 5: (a) ARPES constant-energy intensity plot at the binding energy at 70 meV (energy window of 10 meV) from the same data as Figs. 2 and 3 in the main text. Solid curves guides the contour shape and dashed ones are FC. (b) Virtual Fermi contours estimated by the extrapolation of S2 (thick, red curves) and the actual one from S1 (dashed curves). (c) Another interpretation of FC. A and B correspond to the contour sizes shown in Supplementary Table 1.

| | Semi-minor axis (Å$^{-1}$) | Semi-major axis (Å$^{-1}$) | Area (kT) |
|---|---|---|---|
| Oval | 0.35 | 0.42 | 4.8 |
| A | 0.055 | 0.21 | 0.39 |
| | $k_F$ // $\bar{\Gamma}-\!-\bar{M}$ (Å$^{-1}$) | $k_F$ // $\bar{\Gamma}-\!-\bar{K}$ (Å$^{-1}$) | Area (kT) |
| B | 0.20 | 0.27 | 1.6 |

Supplementary Table I: Sizes of the FCs estimated from the TSS of $SmB_6$(111).

theoretically [7]. To see such phenomena, the fine dispersion of TSS around the crossing points of them, *e.g.* around $\bar{K}$ on $SmB_6$(111), is required. However, such fine analysis is difficult from the current data because of the limited energy resolution. The better resolution, typically an order of 1 meV, is desirable to provide further insight into the overlapping TSS on TKI.

The size of the FCs are also relevant to those observed by dHvA measurements [8–11]. For the comparison, the size of the oval FC is shown in Supplementary Table I. In addition, the overlap of the FCs enables the alternative interpretation of the FCs to be thin ellipsoids and a warped hexagon (A and B in Supplementary Figure 5 (c), respectively). The sizes of them are also shown in Supplementary Table I. For the sake of comparison, the area of them are shown in the kT unit.

Although some values among them apparently agree with those observed by dHvA, *e.g.* 0.4 kT of A with $\beta$ ($\sim$0.3 kT) and $\gamma$ ($\sim$0.4 kT) in ref. [8], one should be careful that it is not yet clear if such comparison could make sense. The TSS observed here and the other ARPES works [12–17] could, in principle, contribute to the electron transport on the crystal surface. In contrast, the Fermi surfaces from dHvA were observed in the condition without bulk electric conduction. Therefore, further discussion is required to understand the agreement of the FC sizes of the TSS observed by ARPES with the FS sizes by dHvA. From experimental aspect, the dHvA measurement around the (111) surface of $SmB_6$ obtained by the similar method to this work would provide useful information for such comparison.

**Supplementary Note 6: Absence of out-of-plane spin polarization along $\bar{\Gamma}$–$\bar{\mathrm{M}}$**

Supplementary Figure 6 shows the spin-resolved energy distribution curves (EDCs) observed at $k_{\mathrm{y}}$ = -0.2 Å$^{-1}$. For this measurement, the polar angle was used (see Supplementary Figure 1 (a)). The intense in-plane spin polarisation shown in Supplementary Figure 6 (a) (the upper panel) agrees well with those shown in Figs. 4 (a) and 4 (c) in the main text. Such agreement independent of the experimental geometry of the SARPES measurement suggests that this spin polarisation is due to the initial states and the spin-dependent photoexcitation process (so-called final-state effect) plays no major role for the spin polarisations observed in this work. The lower panel of Supplementary Figure 4 (a) shows that the out-of-plane spin polarisation is negligibly small. It is natural because the $\bar{\Gamma}$–$\bar{\mathrm{M}}$ line is in the surface mirror plane. On the mirror plane, only the spin polarisation normal to the mirror plane is allowed.

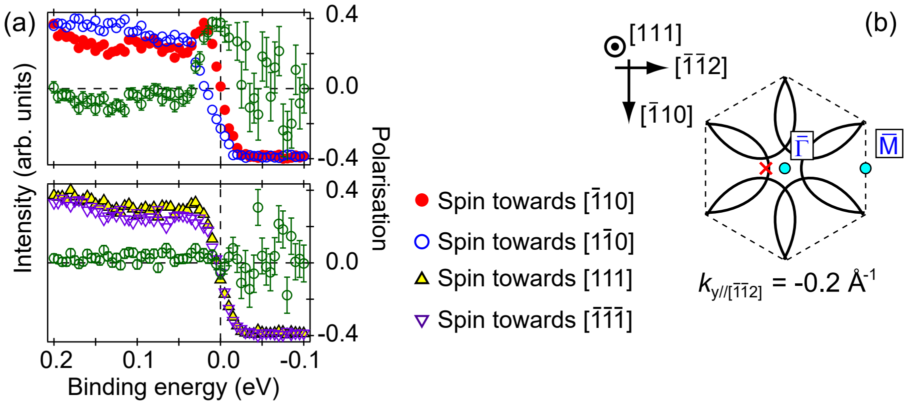


Supplementary Figure 6: (a) SARPES energy distribution curves (EDCs) around $k_{\mathrm{F}}$ together with spin polarisations along the in-plane (the upper panel) and out-of-plane (the lower) orientations. Errors of spin polarisation values are standard statistical errors from photoelectron counting. (b) A schematic drawing of the FC by the metallic surface states. A red cross indicates the position where the spin-resolved EDCs in (a, b) were measured.

**Supplementary Note 7: Spin-resolved ARPES: out-of-plane spins with $C_{3\mathrm{v}}$ surface symmetry**

Supplementary Figure 7 shows the schematic drawings of the influence of the $C_{3\mathrm{v}}$ surface symmetry operations to the out-of-plane spin polarisations of the FCs observed in this work. By the three-fold rotation, a FC around a $\bar{\mathrm{M}}$ point is multiplied to 3 equivalent FCs without any modification to the out-of-plane spin polarisations, as shown in Supplementary Figure 7 (b). On the other hand, the time-inversion operation multiplies a FC by changing the sign of the wave vector $k$ and spin polarisation, as shown in Supplementary Figure 7 (c). The combination of the three-fold rotation and time inversion results in the 6 FCs as shown in Supplementary Figure 7 (d). It should be noted that the out-of-plane spin polarisation along $\bar{\Gamma}$–$\bar{\mathrm{K}}$ is not cancelled out. This spin texture is neither violated by the surface mirror plane, ($\bar{1}10$); all the out-of-plane spins on the FCs changes their signs with respect to the mirror operation from the left to the right sides. The cases are the same for the other two surface mirror planes.

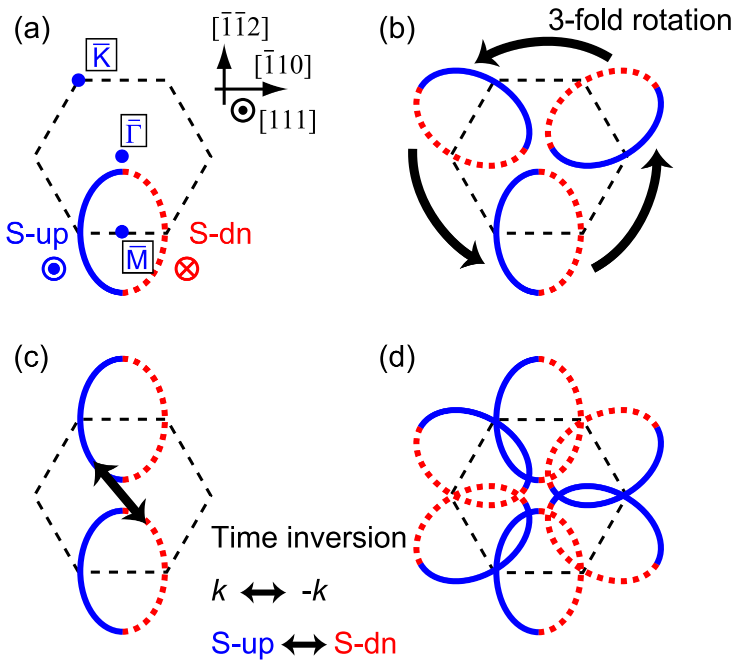


Supplementary Figure 7: Schematic drawings of the out-of-plane spin-polarized FCs with symmetry operations. (a) Single FC at a $\bar{\mathrm{M}}$ point. (b) FCs multiplied by three-fold rotation. (c) FCs multiplied by time inversion. (d) FCs multiplied by the combination between (b) and (c).

## Supplementary Note 8: Detailed analysis of spin-resolved MDCs

In order to discuss the topological order from the surface states at $E_{\mathrm{F}}$, it is enough to exclude the doubly spin-degenerate state, as discussed in the main text. Here, as an additional information, we perform a semi-quantitative analysis of the observed spin-resolved MDCs.

Supplementary Figures 8 (a, b) are for the MDCs at the Fermi level along $\bar{\Gamma}$–$\bar{\mathrm{K}}$. As shown in Supplementary Figure 8 (a), the $I_{tot}$ spectrum along $\bar{\Gamma}$–$\bar{\mathrm{K}}$ has asymmetric peak shape, which makes the analysis along this direction difficult. However, this feature is not only observed by SARPES but also by conventional ARPES, as the red circular markers in Supplementary Figure 8 (a). The slight shift of the peak positions of $I_{tot}$ from SARPES (the solid line in Supplementary Figure 8 (a)) would be due to the wider energy resolution and/or the acceptance angle. To understand it more in detail, we fitted the conventional ARPES MDC by two Gaussians and a third broad feature as indicated by the thin dashed line in Supplementary Figure 8 (a). The peak positions of the Gaussians, $k_{//[\bar{1}10]} = 0.3$ and $0.7$ Å$^{-1}$,

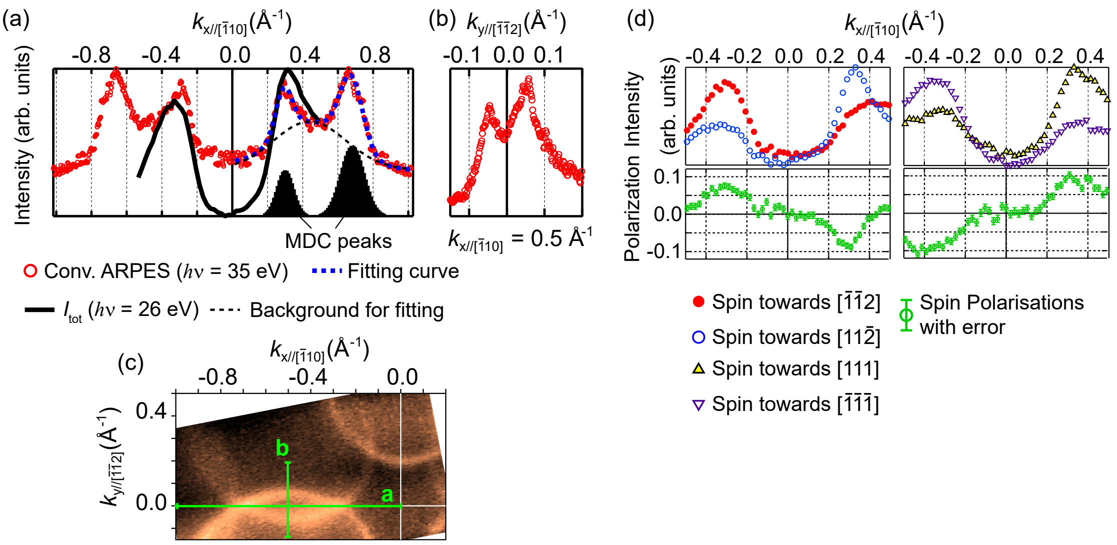


Supplementary Figure 8: (a) Spin-integrated MDCs along $\bar{\Gamma}$–$\bar{K}$ taken at $h\nu$= 35 and 26 eV at the Fermi level (the same data as Supplementary Figure 3 (b) and Fig. 4 (b), respectively). Dashed curves are the fitting curve and its background (see text for details). The MDC at $h\nu$= 35 eV is mirrored with respect to $\bar{\Gamma}$. (b) Spin-integrated MDC taken along [11$\bar{2}$] at $k_{//[\bar{1}10]}$ = 0.5 Å$^{-1}$. (c) ARPES Fermi contour to indicate the region where the MDCs are measured for (a) and (b). (d) SARPES MDCs and spin polarizations measured along $\bar{\Gamma}$–$\bar{K}$ at the Fermi level ($h\nu$= 26 eV). Errors of spin polarisation values are standard statistical errors from photoelectron counting.

are consistent with the metallic surface states shown in Supplementary Figure 3. The origin of the broad background would be due to the FCs lying close to the $\bar{\Gamma}$–$\bar{K}$ line. Actually, the MDC at the Fermi level normal to $\bar{\Gamma}$–$\bar{K}$, as shown in Supplementary Figure 8 (b), indicates two separate peaks corresponding to such FCs, with the finite intensity at $k_{//[11\bar{2}]}$ = 0 Å$^{-1}$ from their tail. Therefore, the asymmetric shape of the spin-resolved MDC shown in Fig. 4 (b) in the main text would be also derived from the overlap of such background and the peak at 0.3 Å$^{-1}$.

Supplementary Figure 8 (d) is the SARPES MDCs and the corresponding spin polarizations of the spin-resolved MDCs. The peaks of the in-plane spin polarizations are clearly around 0.3 Å$^{-1}$, suggesting that this polarization corresponds to the surface states there. The out-of-plane ones does not have such clear peak position. Instead, it has broad intensities at $|k_{//[\bar{1}10]}| > 0.2$ Å$^{-1}$. It would be because both the FC at 0.3 Å$^{-1}$ and the background

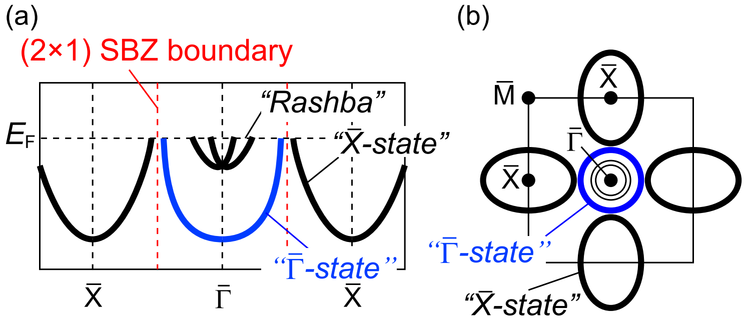


Supplementary Figure 9: Schematic drawings here are based on what were observed in high-resolution ARPES studies [12–17]. Assignments are according to ref. [17]. The thin double-circle around $\bar{\Gamma}$ in (b) is the "Rashba" band in (a).

from the neighbouring FCs are spin polarized along the out-of-plane orientation. At first glance, the spin polarization from the neighbouring FCs might appear to be cancelled out. However, since the $\bar{\Gamma}$–$\bar{\mathrm{K}}$ line is not the mirror plane, the intensities from the neighbouring FCs could be different, as shown in Supplementary Figure 8 (b). Therefore, finite spin polarization from them is expected. Because of such complicated components, the quantitative analysis of the spin-resolved MDCs along $\bar{\Gamma}$–$\bar{\mathrm{K}}$ are quite difficult. However, from the spin polarizations in Supplementary Figure 8 (d), one can see the FC from the surface states has both in-plane and out-of-plane spin components, as stated in the main text. For the quantitative analysis, one needs wider wavevector range as well as the different incident photon energies, polarizations, and the spin polarization to the other orientations. However, such quantitative determination of the photoelectron spin polarization is not the focal point of this research. Note that the spin polarization modulation due to the interference of the photoelectron wavefunctions [18, 19] is also out of the focus of this research.

## Supplementary Note 9: Comparison with the ARPES results on $SmB_6$(001)

We've provided the smoking-gun evidence on the non-trivial topological order of $SmB_6$ based on our spin-integrated and spin-resolved ARPES measurements. Therefore, the surface states on the (001) surface, which has been under debate [12–17] for several years, should also indicate the non-trivial topological order in principle, because the topological order of materials does not depend on the surface orientation but on its bulk electronic structure

and its parities. This problem, the controversial topological assignments on $SmB_6$(001), can be solved by considering the interpretation of the "$\bar{\Gamma}$-state" observed in ref. [17]. Supplementary Figure 9 shows the schematic drawings of the surface band structure observed in earlier ARPES studies [12–17]. In ref. [17], the "$\bar{\Gamma}$-state" band is assigned as a folding of the surface bands surrounding $\bar{X}$ ("$\bar{X}$*–state*") with respect to the (2×1) surface Brillouin zone boundaries. However, the slope of the "$\bar{\Gamma}$-state" band is different from that of its counterpart, as shown in Fig. 2 (c) of ref. [17], suggesting that this state would be a different, individual metallic state. If this state is not an umklapp state but the new state independent of the "$\bar{X}$*–states*", there are three closed FCs on the $SmB_6$(001) surface; one is around $\bar{\Gamma}$ (by the "$\bar{\Gamma}$-state" state) and two are around two $\bar{X}$ points ("$\bar{X}$*–states*"), as depicted in Supplementary Figure 9 (b). Based on this interpretation, one can conclude that there are three closed spin-polarised FCs on both the (111) and (001) surfaces of $SmB_6$, which indisputably indicates its non-trivial topological order. The "Rashba" state observed in ref. [17] (see Supplementary Figure 9 (a)) plays no role in the topological order of the material because it always forms even numbers (2, in this case) of closed FCs around the surface TRIMs. It should be noted that this interpretation is just a possibility based on known results. The detailed origin of the "$\bar{\Gamma}$-state" band would be elucidated by future ARPES and SARPES works with varying incident photon energies and polarisations.

**Supplementary Note 10: Possible origins of electronic states observed around $E_F$ in this work**

Historically, the two mechanisms to form the surface electronic states have been discussed [20]. In a localized-bond picture, surface states can be induced by the surface atomic structure independently from the bulk bands, as the dangling-bond states. The other case is a perturbed-bulkband picture. Surface states are derived from bulk Bloch states, which are perturbed by the truncation of the periodic potential at the surface and consequently localized in near-surface region. In this case, the surface band tends to show the nearly parallel dispersion and similar orbital character to the "mother" bulk bands. Quantum-well states confined in the surface layers, as two-dimensional electron gas states on $SrTiO_3$ [21], also belong to this group. In this context, the surface band $S1$ in this work clearly belongs to the latter case, closely related to the bulk Sm 5d and 4f bands, as evidenced by its dispersion

similar to what is expected to the bulk Sm 5d and 4f bands around $E_{\mathrm{F}}$. If $S2$ and $F$ are also the surface-derived ones, they would also belong to the latter case.

In order to explain the surface electronic structure of $SmB_6$, some theoretical models were discussed. Zhu *et al.* proposed a metallic surface state formed by the combination between the surface polarity and boron dangling bonds at the surface [22]. Based on the classification above, it is not likely a case for $S1$, since the dispersion of the dangling-bond state is completely different from the bulk bands in most cases. Note that a possible surface-band formation based on this mechanism is not excluded. Such state can also be formed away from $E_{\mathrm{F}}$ and it would be the case for $SmB_6$. Actually, the energies of such surface state was calculated to be sensitive to the surface termination condition [22]. The other mechanism was proposed in ref. [17] as "many-body resonance". It was claimed to be originate from the energetic shift of the bulk bands in the surface atomic layers. The "many-body resonance" has similar orbital character and dispersion to its bulk counterparts, but independent from them due to the different boundary condition in the surface atomic layers. Therefore, it is a member of the group of "perturbed bulk bands" and would be a likely case to describe the characteristics of the current electronic states.

It should be noted that all of the proposed mechanisms are compatible to the topological classification. For example, a quantum-well-like state can be a TSS at the same time as simulated in HgTe thin films [23]. The classification procedure merely counts the number of Fermi contours formed by the metallic surface states. As far as the candidate surface state is localized in the surface layers and out of the projected bulk bands, topological classification is always valid, based on the present knowledge. We found no reason to exclude the shifted bulk bands localized in surface atomic layers. Nonetheless, the limitation of the topological classification is discussed in the main text.

---

* Electronic address: `y_oh@fbs.osaka-u.ac.jp`

† Electronic address: `kimura@fbs.osaka-u.ac.jp`

[1] Okuda, T. *et al.* Efficient spin resolved spectroscopy observation machine at Hiroshima Synchrotron Radiation Center. *Rev. Sci. Instrum.* **82**, 103302 (2011).

[2] Okuda, T. *et al.* A double VLEED spin detector for high-resolution three dimensional spin

vectorial analysis of anisotropic Rashba spin splitting, *J. Elec. Spec. Relat. Phenom.* **201**, 23 (2015).

[3] Henk, J., Miyamoto, K. & Donath, M. Retrieving the initial-state spin polarization from spin-resolved photoemission: Proposal for a case study on W(110). *Phys. Rev. B* **98**, 045124 (2018).

[4] Neupane, M. *et al.* Fermi surface topology and hot spot distribution in the Kondo lattice system $CeB_6$. *Phys. Rev. B* **92**, 104420 (2015).

[5] Koitzsch, A. *et al.* Nesting-driven multipolar order in $CeB_6$ from photoemission tomography. *Nature Commun.* **7**, 10876 (2016).

[6] Denlinger, J. D. *et al.* Consistency of Photoemission and Quantum Oscillations for Surface States of $SmB_6$. Preprint at https://arxiv.org/abs/1601.07408 (2016).

[7] Liu, J., Duan, W. & Fu, L. Two types of surface states in topological crystalline insulators. *Phys. Rev. B* **88**, 241303(R) (2013).

[8] Li, G. *et al.* Two-dimensional Fermi surfaces in Kondo insulator $SmB_6$. *Science* **346**, 1208 (2014).

[9] Tan, B. S. *et al.* Unconventional Fermi surface in an insulating state. *Science* **349**, 287 (2015).

[10] Hartstein, M. *et al.* Fermi surface in the absence of a Fermi liquid in the Kondo insulator $SmB_6$, *Nature Phys.* **14**, 166-172 (2018).

[11] Xiang, Z. *et al.* Bulk Rotational Symmetry Breaking in Kondo Insulator $SmB_6$, *Phys. Rev. X* **7**, 031054 (2017).

[12] Miyazaki, H., Hajiri, T., Ito, T., Kunii, S. & Kimura, S. Momentum-dependent hybridization gap and dispersive in-gap state of the Kondo semiconductor $SmB_6$. *Phys. Rev. B* **86**, 075105 (2012).

[13] Xu, N. *et al.* Surface and bulk electronic structure of the strongly correlated system $SmB_6$ and implications for a topological Kondo insulator. *Phys. Rev. B* **88**, 121102 (2013).

[14] Neupane, M. *et al.* Surface electronic structure of the topological Kondo-insulator candidate correlated electron system $SmB_6$. *Nature Commun.* **4**, 2991 (2013).

[15] Jiang, J. *et al.* Observation of possible topological in-gap surface states in the Kondo insulator $SmB_6$ by photoemission. *Nature Commun.* **4**, 3010 (2013).

[16] Xu, N. *et al.* Direct observation of the spin texture in $SmB_6$ as evidence of the topological Kondo insulator. *Nature Commun.* **5**, 4566 (2014).

[17] Hlawenka, P. *et al.* Samarium hexaboride is a trivial surface conductor. *Nature Commun.* **9**, 517 (2018).

[18] Dil, J., H., Meier, F. & Osterwalder, J. Rashba-type spin splitting and spin interference of the Cu(111) surface state at room temperature. *J. Elec. Spec. Relat. Phenom.* **201**, 42 (2015).

[19] Yaji, K. *et al.* Spin-dependent quantum interference in photoemission process from spin-orbit coupled states. *Nature Commun.* **8**, 14588 (2017).

[20] Desjonquères, M., -C. & Spanjaard, D. Concepts in Surface Physics, 2nd ed. *Springer-Verlag, Berlin, Heidelberg* (1996).

[21] Son, W.,-J., Cho, E., Lee, B., Lee, J. & Han, S. Density and spatioal distribution of charge carriers in the intrinsic $n$-type $LaSlO_3$-$SrTiO_3$ interface. *Phys. Rev. B* **79**, 245411 (2009).

[22] Zhu, Z.-H. *et al.* Polarity-Driven Surface Metallicity in $SmB_6$. *Phys. Rev. Lett.* **111**, 216402 (2013).

[23] Luo, J.,-W. & Zunger, A. Design Principles and Coupling Mechanisms in the 2D Quantum Well Topological Insulator HgTe/CdTe. *Phys. Rev. Lett.* **105**, 176805 (2011).
